# The viability of mouse spermatogonial germ cells on a novel scaffold, containing human serum albumin and calcium phosphate nanoparticles

**Published:** 2015-03

**Authors:** Mona Yadegar, Seyed Hossein Hekmatimoghaddam, Saeide Nezami Saridar, Ali Jebali

**Affiliations:** 1 *Department of Biology, East Tehran Branch, Islamic Azad University, Tehran, Iran.*; 2 *Department of Laboratory Sciences, School of Paramedicine, Shahid Sadoughi University of Medical Sciences, Yazd, Iran.*; 3 *Department of Biology, Ashkezar Branch, Islamic Azad University, Ashkezar, Iran.*

**Keywords:** *Cytotoxic effect*, *Scaffold*, *Human serum albumin*, *Calcium phosphate nanoparticles*, *Spermatogonial cell line*

## Abstract

**Background:**

In spermatogenesis, spermatogonial cells differentiate to the haploid gametes. It has been shown that spermatogenesis can be done at in vitro condition. In vitro spermatogenesis may provide an open window to treat male infertility.

**Objective:**

The aim of this study was to evaluate the effects of a novel scaffold containing human serum albumin (HSA)/tri calcium phosphate nanoparticles (TCP NPs) on the mouse spermatogonial cell line (SCL).

**Materials and Methods:**

First, TCP NPs were synthesized by reaction of calcium nitrate and diammonium phosphate at pH 13. Then, serial concentrations of TCP NPs were separately added to 500 mg/mL HSA, and incubated in the 100^o^C water for 30 min. In the next step, each scaffold was cut (2×2mm), placed into sterile well of microplate, and then incubated for 1, 2, and 3 days at 37^o^C with mouse SCL. After incubation, the cytotoxicity of the scaffolds was evaluated by different tests including 3-(4,5-dimethylthiazol-2-yl)-2, 5-diphenyl-tetrazolium bromide (MTT) assay, lactate dehydrogenase (LDH) assay, vital staining, and cell counting. On the other hand, the release of TCP NPs and HSA from the scaffolds was measured.

**Results:**

Based on microscopic observation, the size of cavities for all scaffolds was near 200-500 µm, and the size of TCP NPs was near 50-100 nm. All toxicity tests showed that the increase of TCP concentration in the scaffold did not affect mouse SCL. It means that the percentage of cell viability, LDH release, vital cells, and cell quantity was 85%, 105%, 90%, and 110%, respectively. But, the increase of incubation time led to increase of LDH release (up to 115%) and cell count (up to 115%). Also, little decrease of cell viability and vital cells was seen when incubation time was increased. Here, no release of TCP NPs and HSA was seen after increase of TCP concentration and incubation time.

**Conclusion:**

It can be concluded that the increase of TCP concentration in HSA/ TCP NPs scaffold does not lead to cytotoxicity. On the other hand, the increase of incubation time leads to increase of mouse SCL cell death. In this study, it was found that TCP NPs and HSA could not release from the scaffolds. In future, both proliferation and differentiation of mouse SCL on HSA/TCP NPs scaffold must be checked over more wide incubation times.

## Introduction

Spermatogenesis is a developmental process in which spermatogonial cells differentiate to the haploid gametes (1, 2). Extracellular matrix (ECM) has an important role in the regulation of spermatogenesis in testes. Specially, Sertoli cells are necessary for spermatogenesis (3, 4). In some cases, spermatogenesis process has been stopped, and there are no sperm cells to form zygote. In such cases, spermatogenesis can be done in lab tube. It has been revealed that spermatogenesis can be applied at in vitro condition (5, 6). Nowadays, in vitro spermatogenesis provides an open window to treat male infertility. On the other hand, sperm preservation or cryopreservation of testis is common protocol for some diseases, e.g. pre-pubertal cancer (7). This method has various backwashes and challenges in some patients (8, 9). For these patients, in vitro spermatogenesis is good choice. 

Until now, different artificial ECMs have been described. For the first time, Rafeeqi *et al* used carbon nanotubes (CNTs) scaffold for spermatogonial cell. Based on their microscopic analysis, the cells maintained their shape and function on the scaffold for at least 21 days (10). Another work which was done by Eslahi *et al* showed the effect of poly L-lactic acid scaffold on the mouse spermatogonial stem cell. They indicated that the scaffold significantly increased the formation of cell cluster, compared with control group. Interestingly, they also reported differentiation of spermatogonial stem cells (11). 

Shakeri *et al* investigated behavior of mouse spermatogonial stem-like cells on an electrospun nanofibrillar matrix. They demonstrated that the number of colonies, the number of cells in each colony, and the average area of colonies were increased after culture of cells on the nanofibrillar surfaces for 7 days. They declared that electrospun nanofibrillar surfaces provide a more favorable microenvironment for in vitro short term culture (12). Nano-based scaffolds mimic the architecture of the natural ECM, and provide topographical signals to seeded cells and result in a more physiologically relevant cellular phenotype (      13, 14). Scaffolds provide physical cues for cell orientation and spreading, and remodeling of tissue structure (15). In case of in vitro spermatogenesis on matrixes, although different scaffolds have been investigated, several issues have not been solved. The first issue is low cell viability on the 3D-scaffolds. The second problem is less cell attachment, compared with natural ECM. The third problem is that in the most cases, differentiation does not occur.

Here, the main goal was to synthesize a new scaffold containing human serum albumin (HSA) and tri calcium phosphate nanoparticles (TCP NPs). HSA protein and TCP NPs were used because they are non-toxic and highly biocompatible. Also, many cells can uptake HSA by receptor-mediated endocytosis (16). Importantly, TCP can improve mechanical property when used together with different polymers (17). The second goal was to investigate the effects of the scaffold on mouse spermatogonial cell line (SCL). 3-(4,5- Dimethylthiazol -2)-2,5 diphenyltetrazolium bromide (MTT) assay, lactate dehydrogenase (LDH) assay, vital staining, and cell counting were used in this study.

## Materials and methods

**Materials**

This study was a lab-trial study. It was done in Pajoohesh lab, at summer 2014. Calcium nitrates, diammonium phosphate, HSA, MTT, trypsin containing 5% EDTA, RPMI1640, fetal calf serum (FCS) were provided from Sigma-Aldrich Chemical Co, (St Louis, MO, USA). Isopropanol (70% v/v), trypan blue, and HNO_3_ were sourced from Zyst Fannavar Shargh Company (ZFS Co.), Yazd, Iran. Alamar blue reagent, LDH kit, and CellTiter-Glo Luminescent reagent were purchased from Invitrogen, UK.

**Synthesis of TCP NPs**

First, 36 gr calcium nitrate (Ca (NO_3_)_2_) and 12 gr of diammonium phosphate ((NH_3_)_2_HPO_4_) were dissolved in 525 mL and 375 mL of distilled water (DW), respectively. Then, 25 mL of calcium nitrate were added to 25 mL of diammonium phosphate solution, adjusted to pH 13, and incubated for 6 hr at room temperature. After incubation, the synthesized product was rinsed with DW, and heated at 100^o^C for 30 min. Dried TCP NPs were hardly ball-milled for one hr, and then characterized by scanning electron microscopy (SEM) (S-2400, Hitachi, Japan) and dynamic light scattering (DLS) (ZFS Co., Yazd, Iran) (18).

**Synthesis of HSA/TCP NPs scaffold**

One mL of serial concentrations of TCP NPs (100, 50, 25, 12.5 mg/mL) was separately added to 4 mL of 500 mg/mL HSA, and hardly mixed for 1 min straightaway, HSA/TCP NPs mixture was held in the 100^o^C water and incubated for 30 min. After incubation, HSA/TCP NPs scaffold was freezen at -20^o^C, and then held in the 37^o^C water for 30 min. In this study, four HSA/TCP NPs scaffolds were prepared which their final concentration of TCP NPs was 10, 5, 2.5, and 1.2 mg/mL, respectively. Then, to measure the diameter of the scaffold cavities, they were cut, and observed by an optical microscopy (400x) and by a SEM. In this study, each type of scaffold was cut (2×2 mm), and placed into sterile well of ELISA microplate. For characterization of scaffold cavities, the scaffolds were cut by microtome, stained by Giemsa, and observed by optical microscopy (SaIran, Iran) (18).

**The preparation of mouse SCL suspension**

This type of cell line was provided from Pasteur Institute of Iran. Briefly, 1 mL of RPMI1640 enriched with 10% FCS (RPMI1640-10% FCS) and antibiotic was added to cells, and incubated for 4 days at 37^o^C. After initial incubation, the cells were washed and then transferred to culture flask containing fresh RPMI 1640-10% FCS, and incubated for 14 days at 37^o^C. Since the cells had been adhered to the culture flask, one mL of 100 mg/mL trypsin containing 5% EDTA was added and shaked for 15 min, in order to release of the adhered cells. Then, the cells were rinsed three times with RPMI1640-10% FCS, and the cell suspension was prepared (approximately 10000 cells/mL) (12).

**MTT assay**

First, one piece of each scaffold was incubated with 100 µL of cell suspension for 1, 2, and 3 days at 37^o^C. Then, 50 µL of treated cells and 10 µL MTT were added and incubated at 37^o^C for 3 hr. In the next step, 100 µL of isopropanol (70%) was added to each well, and optical density (OD) of each well was read by ELISA reader (Novin Gostar, Iran) at 490 nm. Finally, the cell viability was measured. The cells which were not exposed to any scaffolds, considered as negative control (19).

**LDH assay**

After incubation of scaffold pieces and the cell suspension for 1, 2, and 3 days, 100 µL of cell suspension was centrifuged at 10000 rpm for 15 min. Then, 10 μL of each supernatant and 1 mL of reagent (lactate and NAD, obtained from LDH kit) was separately added and incubated 5 min at room temperature. After incubation, the mean OD of each sample was read at 340 nm (Novin Gostar, Iran), and then divided to mean OD of control (normalized to control). In the negative control, the cells were not incubated with the scaffolds (20).

**Vital staining**

Same as MTT and LDH assay, the scaffold pieces and cell suspension (100 µL) were separately incubated for 1, 2, and 3 days. Then, 10 µL of 10% trypan blue was added to 10 µL of treated cells, and incubated for 30 min at 25^o^C. Finally, the quantity of live cells (not-stained cells) was recorded by optical microscopy, and the percentage of live cells was calculated. In negative control, the cells were not treated with the scaffolds (20).

**The cell count**

First, each scaffold piece was separately incubated for 1, 2, and 3 days with 100 µL of cell suspension. After incubation, 10 µL of the cell supernatant was held on the Hemocytometer (Marienfeld, Germany), the quantity of the cells was counted by optical microscopy, and then divided to the cell count of control (normalized to control). In the negative control, the cells were not treated with the scaffolds (20).

**The release of TCP NPs and HSA**

Same as other assays, the cell suspension was separately incubated with each scaffold piece for 1, 2, and 3 days at 37^o^C. After incubation, 50 µL of cell suspension was incubated with 50 µL of HNO_3_ for 24 hr, and then centrifuged at 10000 rpm for 15 min. Final, the quantity of Ca^2+^ ions was measured by atomic adsorption spectroscopy, and normalized to control. To measure HSA release, after incubation of the cell suspension with scaffold pieces, the cell suspension was centrifuged at 10000 for 15 min. Then, the supernatant OD of each sample was read at 280 nm (Novin Gostar, Iran), and then normalized to control. The cells which were not exposed to any scaffolds, considered as negative control (18).

**Statistical analysis**

All experiments were done five times, and mean±SD was calculated for each assay. Then, ANOVA (SPSS software, V.16.0 for Windows (SPSS Inc., USA) was used to evaluate the significant differences. In this test, p<0.05 was considered as significant difference.

## Results

**Characterization of TCP NPs and scaffolds**

**Figure 1:**
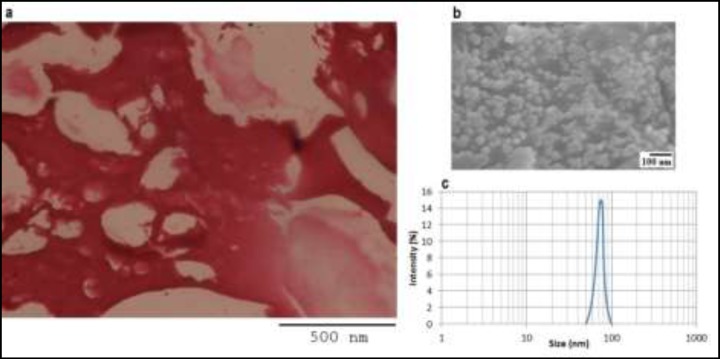
The image of the scaffolds containing 10 mg/mL of TCP NPs, obtained by optical microscopy (a). The microscopic image of TCP NPs, obtained by SEM (b). The size distribution of TCP NPs, obtained by DLS (c).

Based on the observation of scaffold slices by optical microscopy, the size of cavities for all scaffolds was near 200-500 µm (Figure 1a). Since other scaffolds had same structure, their images were omitted. The SEM image and DLS graph of TCP NPs are shown in the Figure 1b and Figure 1c, respectively. The SEM image shows that TCP NPs is approximately spherical, and their size distribution is near 50-100 nm.

**The cytotoxicity results**

**Figure 2:**
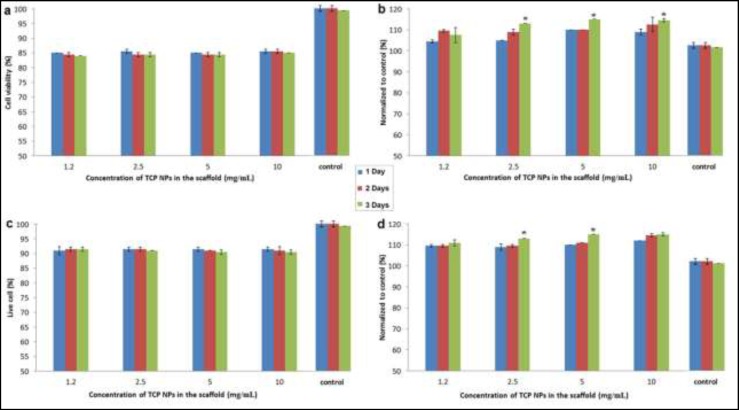
The results of MTT assay (a), LDH assay (b), vital staining (c), and cell counting (d). All tests were done 5 times (n=5), and then normalized to control. * P<0.05 compared with the value related to one week incubation at same concentration.

The results of MTT assay, LDH assay, vital staining, and cell counting are shown in the Figure 2a, Figure 2b, Figure 2c, and Figure 2d, respectively. As seen at all of them, the concentration of TCP NPs in the scaffolds could not affect the results. But, the increase of incubation time led to few increase of LDH release and cell count, and few decrease of cell viability and vital cells. The significant differences between different groups are indicated in the figures.

**The release results**

**Figure 3:**
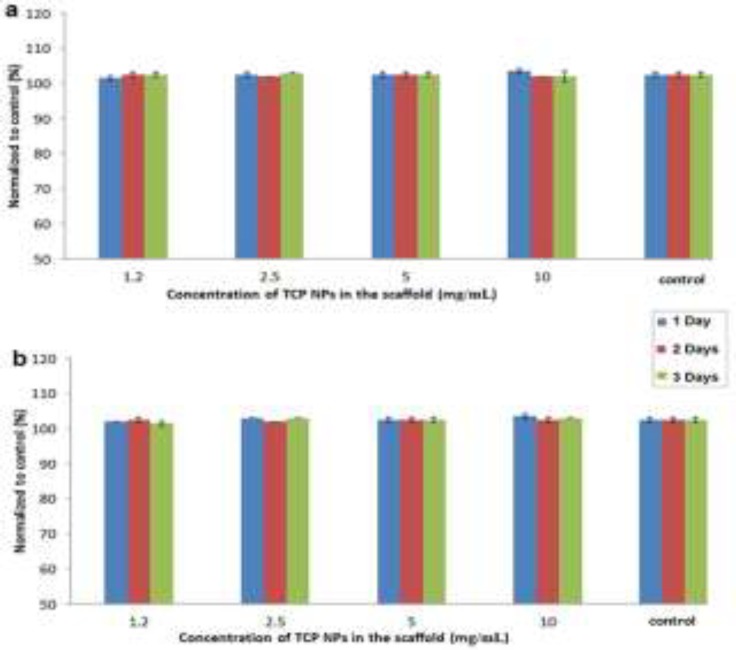
The release of TCP NPs (a) and HSA (b) from the scaffolds at different incubation times. All tests were done 5 times (n=5), and then normalized to control.

The release of TCP NPs and HSA is shown in the Figure 3a and Figure 3b, respectively. No release of TCP NPs and HSA was seen after increase of TCP concentration and incubation time. Furthermore, no significant differences were observed between different groups.

## Discussion

In this study, HSA/TCP NPs scaffold was synthesized, and its cytotoxic effects on the mouse SCL were investigated. Here, different tests including MTT assay, LDH assay, vital staining, and cell counting were used to show the effects of HSA/TCP NPs scaffold on mouse SCL. The main logics of this study were: 1) designing of a new scaffold based on natural components, 2) evaluating of the scaffold on the mouse SCL, and 3) using of HSA/TCP NPs scaffold in future for male infertility. 

The results of all tests revealed two important findings: The first important finding was that the increase of TCP NPs in the scaffold did not lead to change of cytotoxicity. The authors explain that the result is due to powerful binding of TCP NPs and HSA in the scaffold. This phenomenon is confirmed by the release test. As mentioned, no release of TCP NPs and HSA was observed after increase of concentration and incubation time. The authors think that the higher concentrations than 10 mg/mL must be tested in the future study. The reason of usage of these concentrations is addressed to initial concentration of TCP NPs, i.e., we could not prepare the suspension of TCP NPs higher than 100 mg/mL because of rapid precipitation. Supervising, increase of TCP concentration not only did not affect the result of cytotoxicity and release tests, but also did not change the strength (solidity) appearance.

The second important finding was that the increase of incubation time led to few increase of LDH release and cell proliferation, and few decrease of cell viability and vital cells. Overall, these results showed that the more incubation time, the higher cytotoxic effects. It seems that the bad environmental and nutritional conditions are the reason of the higher cytotoxic effects when SCL incubated more times with scaffolds. Note, this pattern is common in the cell culture, and is not a supervising result (10, 11). In the future studies, the cells must be incubated and evaluated more than three days. Also, other complementary tests such as electron microscopy, molecular assays, etc. must be considered. On the other hand, some in vivo biocompatibility tests including hemolysis, irritation, sensitization, and implantation must be added, in order to complete data (-).

In this study, we used HSA because of its several advantages including natural nature, good biocompatibility, and high adsorption property. It has been demonstrated that HSA could take up by different cells as a food source (24). Also, it has been shown that albumin can be covalently labeled with a drug or a fluorescent dye, and can be enter to human cells (16). Albumin is taken up by receptor-mediated endocytosis and degraded into the lysosome. Interestingly, albondin which is endothelia gp60 protein can bind to albumin, and leads to transcytosis (25). Kuchar *et al* used the albumin-binding domain expressed by gram-positive bacteria as a part of scaffold. Not only, albumin-binding domain is important for bacteria, but also can be used for protein purification and immobilization (26). 

Nseir et al designed electrospun albumin scaffold as an artificial tissues, and showed improved elasticity of the scaffold. It was capable to adsorb different serum proteins, e.g. laminin, which led to high cell-matrix interactions. They suggested that the electrospun albumin scaffold could be chemically modified by various biomolecules for different applications (27). Also, Jaklenec *et al* made a novel scaffold from albumin-loaded microspheres for tissue engineering. They showed a good compatibility of the scaffolds when incubated with fibroblast cells (28). Ferrero-Gutierrez  designed a novel HSA scaffold for improvement of spinal cord injury. The scaffold seeded adipose derived stem cells (ADSCs) and olfactory ensheathing cells (OECs). 

It was revealed that ADSCs and OECs adhered to the scaffold, and had no toxicity (29). Luisi *et al* developed an albumin-derived peptide scaffold which was from the sequence of the IIA binding site of HSA. They revealed that the scaffold could bind to various molecules and had catalytic properties (30). Gallego *et al* investigated the biocompatibility of a novel albumin scaffold. The scaffold was a favorable substrate for the growth and differentiation of osteoblasts and was a good candidate for bone tissue engineering (31, 32). 

Furthermore, Weszl *et al* showed mineralized scaffolds could be used as a bone graft. They implied that although the coating of scaffolds with fibronectin and collagen improved the seeding efficiency, HSA led to remarkable seeding and proliferation (33). In this study, TCP was used, because it is biocompatible and biodegradable (17). TCP leads to low mechanical strength and adsorption capacity, when used alone. But the using of different polymers such as chitosan, poly (lactic-co-glycolic acid (PLGA), Methoxymethamphetamine, polypropylene, and polycaprolactone with TCP improves its mechanical and chemical properties (17). Al-Munajjed *et al*, Keeney *et al*, and Inzana *et al* worked on development of a collagen/TCP scaffold as a bone substitute. They showed increased mechanical stiffness, maximal biocompatibility, and highly osteoconductivity (-). 

Lickorisha *et al* synthesized a polyester/TCP scaffold for bone tissue repair. They presented the third generation of scaffold with enhanced in vitro and in vivo performances (37). Yang *et al* prepared PLGA/TCP scaffolds, in order to controllable release of dexamethasone (Dex) and BSA. Based on their results, controllable dual-release was seen by the PLGA/TCP scaffold (38). Ribeiro *et al* synthesized TCP porous scaffold by albumin as a foam generating agent. They indicated that a variety of scaffold geometries has been prepared by the heating of HSA (39). In case of spermatogonial cells, various scaffolds have been introduced. Rafeeqi *et al* used CNTs as a scaffold. They showed the cells maintained their shape and function on the scaffold for at least 21 days (10). 

Eslahi *et al* showed the effect of poly L-lactic acid scaffold on the mouse spermatogonial stem cell. They revealed the scaffold increased formation of cell cluster and differentiation of spermatogonial stem cells (11). Shakeri *et al* investigated electrospun nanofibrillar scaffolds. They showed increase number of colonies, the number of cells in each colony, and the average area of colonies after 7 days (12). Jebali *et al* designed a novel scaffold based on HSA and hydroxyapatite NPs. They showed the scaffold had no cytotoxic effects on mouse SCL (18).

## Conclusion

Taken together, this study demonstrated that HSA/TCP NPs scaffold could be easily synthesized, and has no remarkably cytotoxic effects on mouse SCL. It can be concluded that the increase of TCP concentration in HSA/TCP NPs scaffold does not affect its cytotoxicity. Moreover, the increase of incubation time led to increase of cell death. Here, it was found that TCP NPs and HSA could not release from the scaffolds. HSA/TCP NPs scaffold must be used in vivo, in order to evaluate its efficacy at actual conditions. Certainly, the proliferation and differentiation of human spermatogonial cells must be checked over different incubation times. In the future, the scaffold may be beneficial to treat male infertility.

## Note

***This article extracted from M.Sc. thesis. (Mona Yadegar)***
